# Bistable Electrical Switching Using a Crown Ether-Based Monolayer Electrolyte on WSe_2_ Field-Effect Transistors with Various Salts

**DOI:** 10.1021/acsaenm.4c00799

**Published:** 2025-01-30

**Authors:** Huiran Wang, Shubham Sukumar Awate, Susan K. Fullerton-Shirey

**Affiliations:** † Department of Chemical and Petroleum Engineering, University of Pittsburgh, 3700 O’Hara Street, Pittsburgh 15260, United States; ‡ Department of Electrical and Computer Engineering, University of Pittsburgh, 3700 O’Hara Street, Pittsburgh 15260, United States

**Keywords:** two-dimensional materials, atomic force microscope, electric double layer, nonvolatile memory, iontronics, field effect transistor, monolayer electrolyte

## Abstract

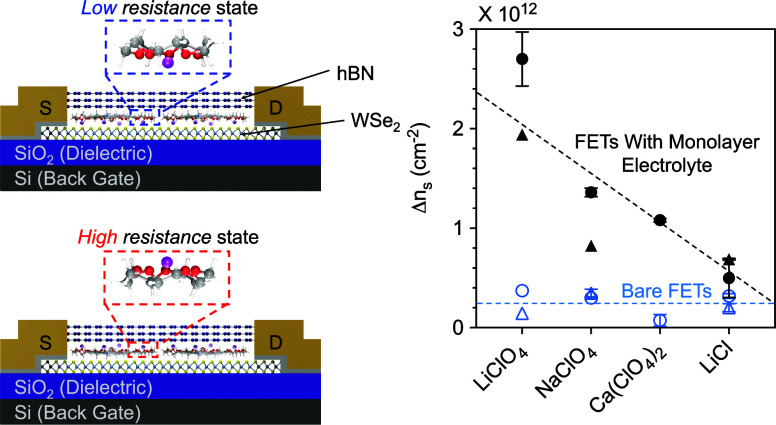

Bistable electrical switching using a crown-ether-based electrolyte on WSe_2_ field-effect transistors (FETs) is measured for four salts: LiClO_4_, NaClO_4_, Ca(ClO_4_)_2_, and LiCl. The solid-state monolayer electrolyte comprises cobalt crown ether phthalocyanine in which cations are solvated by 15-crown-5 ethers. The switching mechanism is the toggling of cations through the crown ether cavity in response to an applied field, creating low and high resistance states in the WSe_2_ channel. This work shows that bistability is not unique to Li^+^ and extends to other perchlorate-based salts with Na^+^ and Ca^2+^ cations. LiClO_4_ induces the largest sheet density (2 × 10^12^ cm^–2^) followed by Ca(ClO_4_)_2_ (1 × 10^12^ cm^–2^) and NaClO_4_ (0.8 × 10^12^ cm^–2^). The impact of the anion was evaluated by replacing LiClO_4_ with LiI and LiCl. A homogeneous deposition of LiI could not be achieved, and LiCl only induced 0.2 × 10^12^ cm^–2^—an order of magnitude less charge than the perchlorate-based salts. Devices with LiCl required the largest voltages to achieve switching and had the smallest ON/OFF ratio in a 6 h state retention test. The results point to the anion playing a critical role in bistability, and Li^+^ as the best performing cation in terms of doping density, minimum switching voltage, and state retention.

## Introduction

Crown ethers are macrocyclic polyethers with a molecular repeat unit of (−CH_2_CH_2_O−)_*n*_, where the size of the cavity increases with increasing *n*. The naming convention (e.g., 15-crown-5, or 15C5) indicates the number of carbon atoms followed by oxygen atoms in the ring.^1^ Crown ethers have a wide range of applications, including in drug delivery as an encapsulant^2^ and permeability enhancer,^3^ as a chemical sensor for metal ion detection,^4−6^ and as a building block for ion-conducting channels.^7,8^ These applications are enabled by the chelation of cations with crown ethers through interactions with the electronegative ether oxygens. When crown ethers are tethered to a large and flat anchoring molecule, such as a phthalocyanine (Pc), they can lay flat on a surface in an ordered monolayer. That is, the molecules are arranged in an array with the plane of the crown ether ring parallel to the substrate. Such a crown-ether functionalized monolayer has been demonstrated on gold by dip coating,^9,10^ and on two-dimensional (2D) crystals—including graphene—by drop-casting and annealing.^11^

Because crown ether Pc molecules can form an ordered monolayer on the surface of 2D materials, it motivates the exploration of their use in 2D heterostacks, including field-effect transistors (FETs). In our previous work, we drop-cast cobalt crown ether phthalocyanine (CoCrPc) and LiClO_4_, referred to as a “monolayer electrolyte” onto 2D crystals. The molecularly thin electrolyte is solid state, electrically insulating,^11,12^ and lays in a flat, ordered array of 0.5 nm thickness on 2D crystals.^11^ Yoshimoto and coworkers showed that when the monolayer CoCrPc is exposed to a salt solution, and the solvent is evaporated, the cations preferentially bind with the crown ethers.^10^ We showed that upon backgating, the monolayer electrolyte induces nonvolatile channel doping of graphene,^13^ MoS_2_,^12^ and WSe_2_^12^ due to ion-gating. The mechanism, supported by density functional theory (DFT) calculations, involves the formation of electric double layers. Specifically, the cations are stabilized in two states: near and far from the channel. Each crown ether cavity, which is aligned parallel to the channel, presents an energy barrier to a cation passing through in response to an applied electric field.^14^ When a negative bias is applied to the backgate, the cations are pulled near the channel surface, inducing image charge that n-type dopes the channel and provides a low-resistance state, as detected by a shift in the Dirac point of graphene,^13^ and a shift in the threshold voltage of WSe_2_ and MoS_2_.^12^ This state is retained in the absence of an applied bias. When a positive bias is applied to the backgate, the cations are driven away from the channel surface, creating a high-resistance state, and a p-type shift is detected. The two states are stabilized by the energy barrier presented to the cation by the crown ether, which disallows further ion transport through the cavity in the absence of an applied field, giving rise to nonvolatility. With a crown ether to Li^+^ molar ratio of 4:1, charge densities on the order of 10^12^ cm^–2^ are induced in graphene,^13^ and WSe_2_.^12^ For WSe_2_ FETs, the ON/OFF ratio exceeds 10^4^ at 0 V read, the device remains stable after 1000 cycles, and the retention time for each state exceeds 6 h, which was the maximum time measured.

It is reasonable to assume that different cationic species will have distinct coordination energies with the crown ether, and will therefore present unique energy barriers to pass through the 15C5 cavity under an applied field. This is intriguing because multiple barriers to switching could allow application of this material in multibit information storage devices. In selecting possible cations, two conditions must be met: 1) the crown ether cavity size needs to be sufficiently large^15^ for an ion to pass through, and 2) the equilibrium constant between the crown ether and ion needs to be sufficiently strong to create the complex,^16^ but weak enough to allow the ion to pass through the cavity in response to an applied field. DFT calculations suggest that Na^+^ and Ca^2+^ meet these criteria.^14^ Thus, in this work, we explore additional salts—NaClO_4_, Ca(ClO_4_)_2_, and LiCl—and discover that Li^+^ is not the only cation that induces bistability in the monolayer electrolyte—both Na^+^ and Ca^2+^ do as well. We also show that 15C5-CoCrPc:LiClO_4_ is the best performing electrolyte in terms of charge density, switching voltage, and ON/OFF state retention, while LiCl is the worst performer. Two distinguishable states can persist for at least 6 h for all complexes containing perchlorate anions, while LiCl demonstrates a state retention of less than 10 min, highlighting the important role of anions in the switching mechanism.

## Results and Discussion

To determine the extent to which the identity and valency of the ions, and the identity of the casting solvent impacts the quality of the deposition, atomic force microscopy (AFM) measurements are made. The monolayer electrolyte is prepared on WSe_2_ by drop-casting and annealing 15C5-CoCrPc, followed by drop casting LiClO_4_, NaClO_4_, Ca(ClO_4_)_2_, LiCl and LiI from ethanol. The molar ratio of CoCrPc to cations is 1:1, which equals a crown ether to cation molar ratio of 4:1, the same as our previous work.^12^ These 5 salts are chosen because they are soluble in solvents with high vapor pressures, the cations fit through the crown ethers,^14^ and the cations bind to the crown but not so strongly that they cannot pass through the cavity.^14^ Three of the five share a common cation (Li^+^) and chemically distinct anions (ClO_4_^–^, Cl^–^ and I^–^), and three share a common anion (ClO_4_^–^) and chemically distinct cations (Li^+^, Na^+^ and Ca^2+^). Using this approach, the impact of chemical identity on performance can be evaluated. The thickness of the CoCrPc on WSe_2_ is ∼0.5 nm, as measured on multiple flakes on multiple locations by AFM and reported in Figure S1. This thickness is consistent with our prior measurements.^11−13^ To compare across solvents, LiClO_4_ is also deposited from acetone and acetonitrile. Exposure of the monolayer electrolyte to moisture, including the moisture in air, can disrupt the morphology of the monolayer electrolyte within few minutes;^11^ therefore, solution preparation, drop-casting, annealing, and AFM measurements are performed inside an argon-filled glovebox.

**Figure 1:**
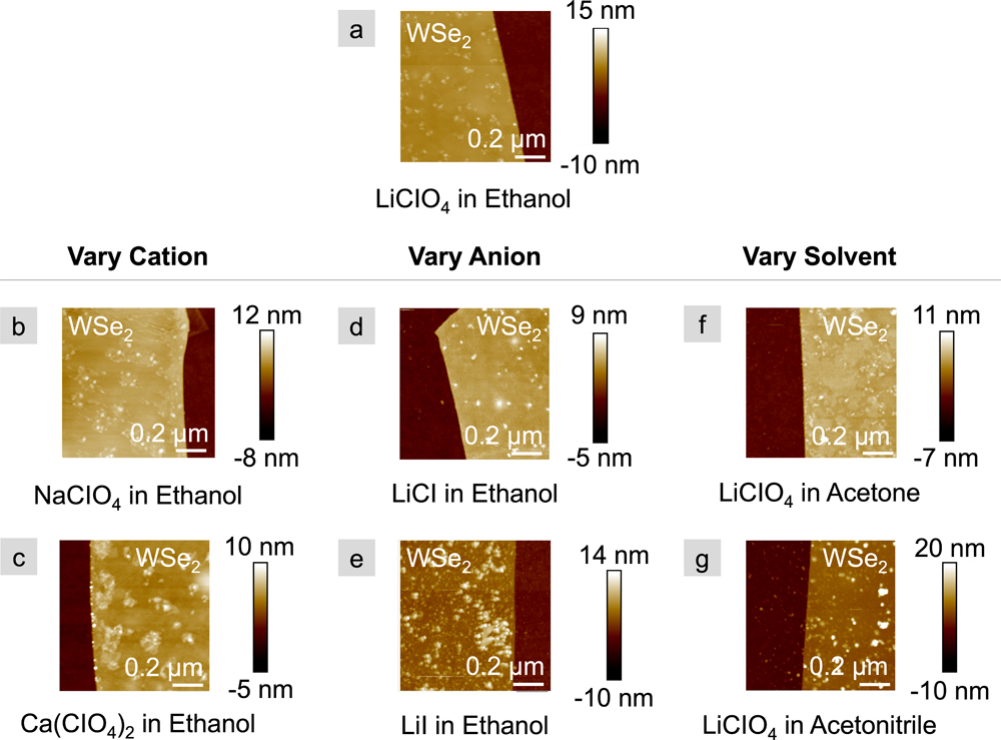
AFM scans of monolayer electrolytes on exfoliated WSe_2_ flakes. 15C5-CoCrPc and (a) LiClO_4_, (b) NaClO_4_, (c) Ca(ClO_4_)_2_, (d) LiCl and (e) LiI, cast in ethanol; and LiClO_4_ cast in (f) acetone and (g) acetonitrile. WSe_2_ flakes were exfoliated on 90 nm SiO_2_/Si substrates, and the electrolytes are drop-cast and annealed in an argon-filled glovebox.

**Figure 2:**
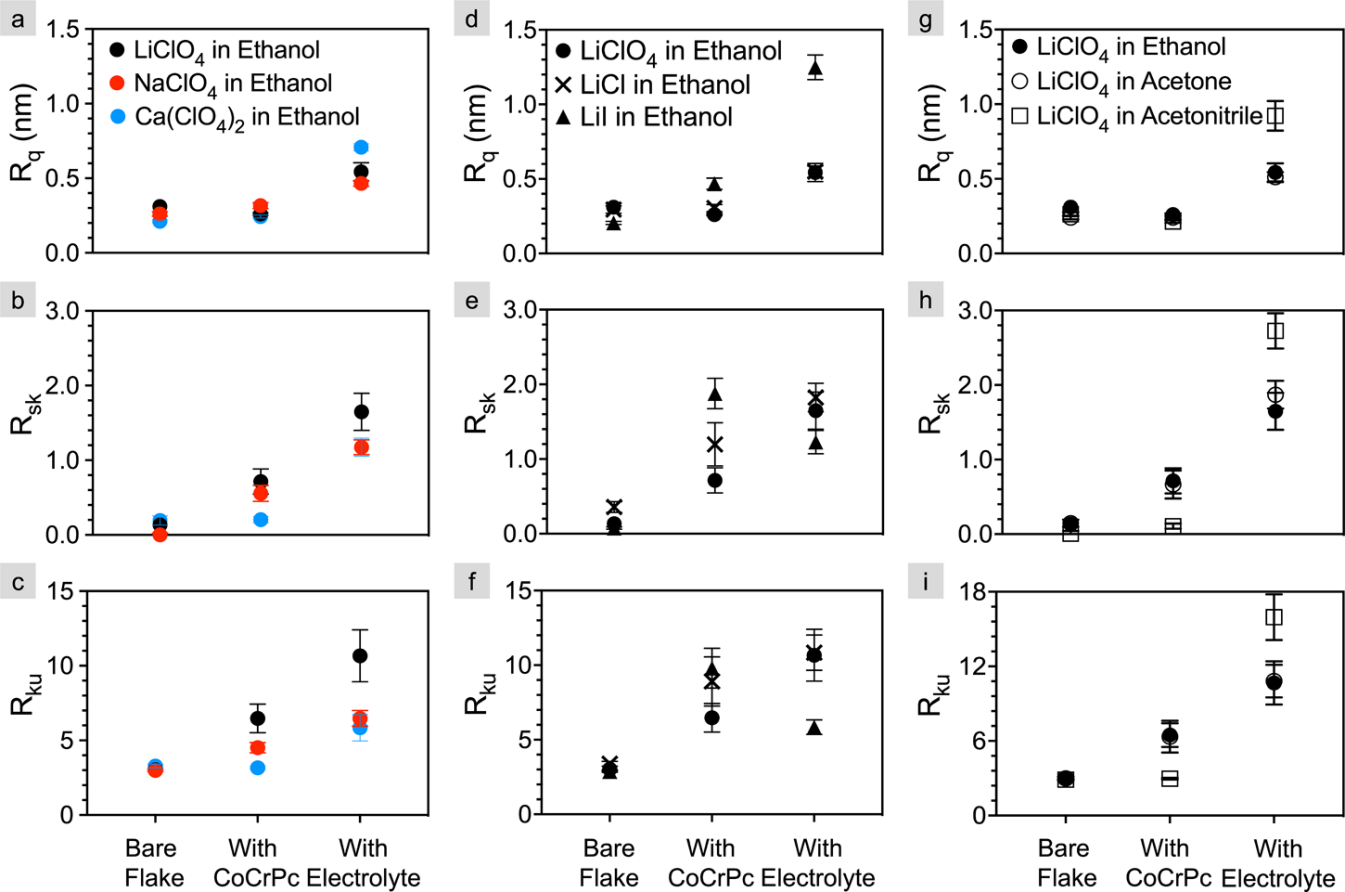
Topographical amplitude parameters of bare flakes, 15C5-CoCrPc only and 15C5-CoCrPc with five salts drop-cast using three solvents (i.e., “with electrolyte”). R_*q*_ is shown in the top row (a, d, g), R_*sk*_ in the middle row (b, e, h) and R_*ku*_ in the bottom row (c, f, i). Data for LiClO_4_ drop cast with ethanol is included in all sub-figures to aid comparison. Cations are varied in column one from Li^+^ to Na^+^ and Ca^2+^ (a, b, c), anions from ClO_4_^–^ to Cl^–^ and I^–^ (d, e, f) and solvents in column three from ethanol to acetone and acetonitrile (g, h, i). Each data point represents the average across four to six flakes of six, 200 by 200 nm scan areas per flake. The error bars represent the standard error of the mean.

AFM scans of the monolayer electrolytes are shown in Figure 1. The previously studied combination of 15C5-CoCrPc with LiClO_4_ in ethanol is highlighted in Figure 1a. The cation is varied from Li^+^ to Na^+^ and Ca^2+^ in the left column, while the anion and solvent remain unchanged (Figure 1a–c). The anion is varied from ClO_4_^–^ to Cl^–^ and I^–^ in the middle column, while the cation and solvent remain unchanged (Figure 1a,d,e). Finally, the solvent is varied from ethanol to acetone to acetonitrile in the right column, while the salt remains LiClO_4_ (Figure 1a,f,g). While aggregates appear in the scan regions for all monolayer electrolytes, their size and density vary. To quantify these variations, the root-mean-square roughness (R_*q*_), skewness (R_*sk*_), and kurtosis (R_*ku*_) are calculated from the average of six, 200 by 200 nm locations across each of four to six WSe_2_ flakes. The average of all measurements on all flakes along with the standard error of the mean are reported in Figure 2. Note that R_*sk*_ is the first derivative of the surface roughness, the sign of which indicates a preponderance of peaks (positive) or valleys (negative). R_*ku*_, the fourth moment of the surface roughness, captures the sharpness of those peaks or valleys.^17−19^ R_*q*_, R_*sk*_, and R_*ku*_ are reported for bare WSe_2_ flakes, 15C5-CoCrPc-only, and electrolyte (i.e., 15C5-CoCrPc and ions). In general, the 15C5-CoCrPc does not increase the surface roughness significantly from that of the bare flake; however, whatever roughness that develops is in the form of peaks, as indicated by a positive R_*sk*_.

The addition of the salt disrupts some of the order of the 15C5-CoCrPc monolayer, increasing R_*q*_ by a factor of 2 in most cases, but up to a factor of 4 to five for LiClO_4_ in acetonirtile and LiI in ethanol, respectively. Correspondingly, similar trends are observed for R_*sk*_ and R_*ku*_ when the salt is introduced. That is, the roughness is in the form of peaks that become more pronounced and sharper with the addition of salt.

Because the combination of CoCrPc and LiClO_4_ cast from ethanol as a monolayer with few, nanometer-sized aggregates, gave favorable, nonvolatile memory characteristics in our previous work,^11−13^ we regard monolayer electrolytes with R_*q*_, R_*sk*_ and R_*ku*_ values equal to or better than those of CoCrPc and LiClO_4_ in ethanol to be indicative of a good quality deposition. Specifically, R_*q*_ < 0.8 nm, R*_sk_<* 2, and R_*ku*_ < 10. These selection criteria also ensure exclusion of electrolytes that result in large aggregates (i.e., > 20 nm) because these would prohibit 2D material stacking with high quality interfaces. Thus, NaClO_4_, Ca(ClO_4_)_2_, and LiCl in ethanol are chosen for electrical measurements, and LiI in ethanol and LiClO_4_ in acetone and acetonitrile are eliminated from further consideration.

**Figure 3:**
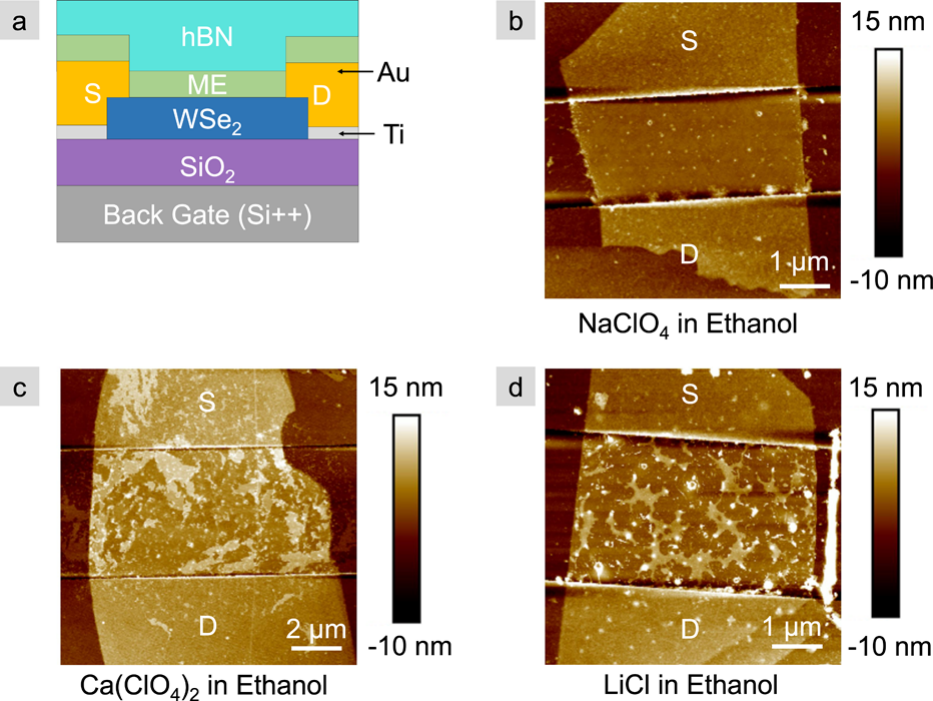
Device schematic and channel characterization by AFM. (a) Cross-sectional schematic of the monolayer electrolyte-coated and h-BN capped WSe_2_ FETs. AFM scans of WSe_2_ FET channels after drop-casting with CoCrPc and (b) Na(ClO)_4_, (c) Ca(ClO_4_)_2_, and (d) LiCl in ethanol. Note, AFM measurements were made prior to capping with h-BN.

Each of the down-selected monolayer electrolytes are deposited on WSe_2_ FETs to test for doping, bistaibility, retention, and minimum switching voltage via backgating. To minimize device-to-device variability, the number of WSe_2_ layers in all devices is between 5 to 12. A cross-sectional schematic of the device is shown in Figure 3a, which includes an h-BN capping layer. We previously showed that this monolayer electrolyte/h-BN interface is essential to stabilize the OFF state of the device.^12^ AFM scans of the FET channels onto which the three selected electrolytes are deposited are shown in Figure 3b–d. Note that the deposition quality on the FET is worse than the bare flakes in Figure 1, in particular for Ca(ClO_4_)_2_ and LiCl, shown in Figure 3c,d. This is due, in part, to the underlying roughness originating from lithographic resist residue^19^ that is amplified by the subsequent deposition of the salt. Although contact mode AFM cleaning is used on each channel, remnants of the residue remain. The presence of the metal contacts, and the different surface chemistry between the 2D crystal and the metal also serve to disrupt the homogeneity of the electrolytes such that deposition on FETs is less homogeneous than bare flakes.

**Figure 4:**
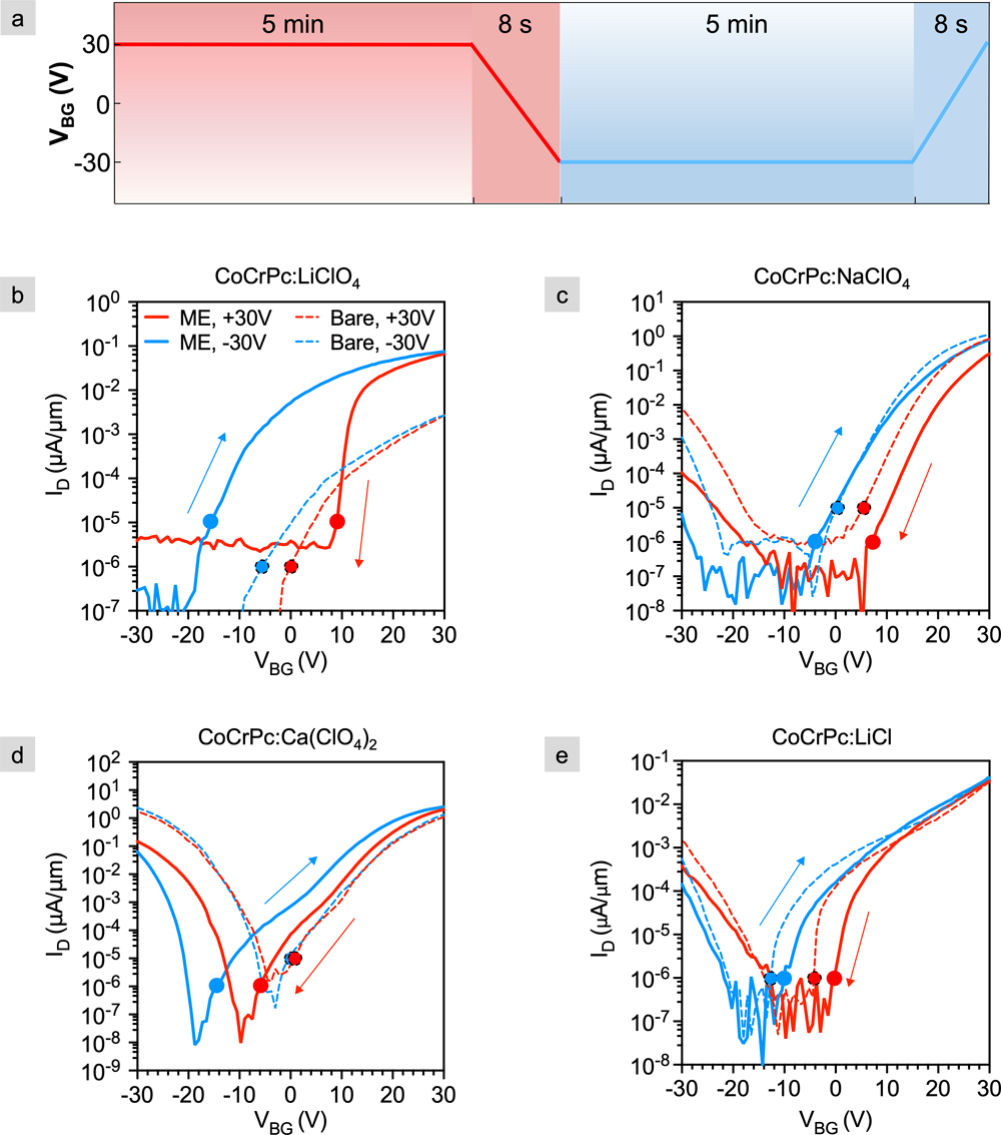
Transfer measurements of programmed bare (dashed) and programmed h-BN-capped monolayer electrolyte (ME) devices (solid). (a) Programming and sensing protocol. A 5 min, +30 V programming (red) is followed by a single-sweep transfer measurement to sense the OFF-state programming. A 5 min, –30 V programming (blue) is followed by a single sweep transfer measurement to sense the ON-state programming (sweep rate ∼12–13 V/s for programming of both states). Transfer measurements for 15C5-CoCrPc with (b) LiClO_4_ (1 – D1, data from Liang et. al.,^12^) (c) NaClO_4_ (2 – D1), (d) Ca(ClO_4_)_2_ (3 – D1), and (e) LiCl (4 – D1) cast from ethanol. V_*DS*_ equals 500 mV, and the sweep rate for LiClO_4_ is 10 V/s while the rest of the salts are 7.5 V/s. The subtheshold voltage, V_*S*_ is indicated by the circles.

The extent to which the monolayer electrolytes dope the channel is measured for the original electrolyte (15C5-CoCrPc and LiClO_4_) and the three newly identified electrolytes by back-gated transfer measurements following the write/erase and read protocol shown in Figure 4a. Note that a three-terminal device is used so that the gate can be used exclusively to set the state of the device ON or OFF while the source and drain terminals sense the state. To set the OFF state, V_*BG*_ is held at +30 V for 5 min. Then, a single sweep measurement is taken from V_*BG*_ = +30 to −30 V to sense the programmed OFF state. Similarly, to set the ON state, V_*BG*_ is set to −30 V for 5 min, followed by a single sweep from V_*BG*_ = −30 to +30 V to sense the state. Note that 30 V is dropping through 90 nm of backgate oxide and then being screened by the doped WSe_2_, suggesting that only a small fraction of the applied voltage reaches the electrolyte. V_*DS*_ equals 500 mV, and the sweep rate is ∼13 V/s. Note that 5 min of programming is chosen so that these data can be directly compared to those of Figure 2 in our previous publication focused on 15C5-CoCrPc and LiClO_4_.^12^

Focusing first on the transfer characteristics of the bare FETs (dashed lines) in Figure 4, there exists some change in the doping density of the channel after 5 min programming even without the electrolyte, as indicated by the hysteresis in the forward and reversed sweeps. This is common for bare FETs due to electron/hole trapping.^20^ However, the hysteresis, and therefore the extent of doping, increases substantially with the addition of the monolayer electrolyte/h-BN to the bare flakes for all salts measured. The largest hysteresis occurs in CoCrPc:LiClO_4_ for which the ON current also increases significantly.

To isolate the impact of programming on the transfer measurements from those without programming, three consecutive double-sweep transfer measurements are collected, followed by three single sweep measurements after programming at each polarity for both bare and electrolyte-coated FETs (Figures S2 and S3, respectively). The repeatability of the transfer measurements and the absence of any peaks or valleys in the drain current suggests that additional mechanisms (e.g., redox reactions) are not present or significant. To capture device-to-device variability, and variability that inhomogeneous electrolyte coverage may induce, each electrolyte is characterized on two devices each on separate chips–except Ca(ClO_4_)_2_ for which there is only one device measured. The transfer measurements in Figures S2 and S3 provide additional support that the monolayer electrolyte provides significantly more doping than the bare FETs, and this is true for all measured electrolytes. Thus, we can conclude that the combination of 15C5-CoCrPc and LiClO_4_ is not the only monolayer electrolyte that can dope a 2D semiconductor; however, the extent of doping varies with the identity of the salt.

**Figure 5:**
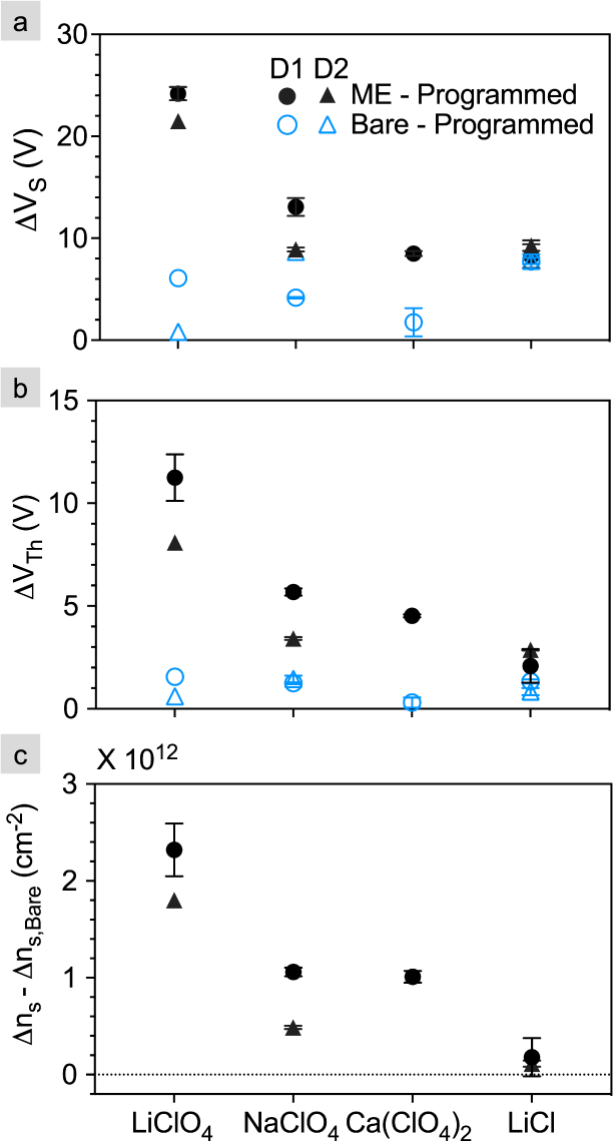
Difference in (a) the subthreshold voltage (ΔV_*S*_), (b) the threshold voltage (ΔV_*Th*_), and (c) corresponding change in the carrier density (Δn_*S*_ – Δn*_S,Bare_*) after 5 min programming. D1 and D2 stand for Devices 1 and 2. For most devices, data are averaged over three ON/OFF programming cycles, and error bars represent one standard deviation from the mean. The blue unfilled symbols in (a) and (b) stand for data from their corresponding bare devices.

To quantify the extent of doping across salts and devices, the n-branch of all transfer curves is chosen for further analysis as it has the largest ON/OFF ratio in the window of the measurement. Although the monolayer electrolyte devices share some similarity with ferroelectric random access memory in terms of polarization leading to a hysteresis, the switching mechanism is unique and the same terminology cannot be applied. Instead, we track the minimum subthreshold voltage of the n-branch, V_*S*_, and indicate its location by circles in Figure 4. Specifically, the data in the subthreshold region is fit to a line over two decades in current, and the voltage on the fit line that corresponds to 10^–5^ μA/μm for LiClO_4_, and 10^–6^ μA/μm for all other salts is defined as V_*S*_. ΔV_*S*_ is defined as the difference between the ON and OFF V_*S*_, and it captures the extent of hysteresis. The average ΔV_*S*_ values are reported in Figure 5a for all four monolayer electrolytes where the open (filled) symbols represent the ΔV_*S*_ in the bare (electrolyte) devices. The shape of the symbols indicates the device: device 1 (D1) and device 2 (D2). All data are averaged across three measurements on D1 and D2 in Tables S1 and S2. The only salt for which there is no device with a statistically significant difference in ΔV_*S*_ between the bare and monolayer electrolyte devices is LiCl, meaning that LiCl is the only salt for which there is no significant hysteresis, and therefore no switching.

To estimate the change in sheet carrier density (Δn_*S*_) on programming, V_*Th*_ is extracted via a linear fit of the n-branch over two decades in current. Linear transfer plots with V_*Th*_ indicated are provided in Figures S2 and S3 for the bare and monolayer electrolyte FETs, respectively, and V_*Th*_ is tabulated in Tables S3 and S4 for all bare and monolayer electrolyte FETs, respectively. ΔV_*Th*_ is averaged for each salt across all measurements of all devices, and summarized in Figure 5b. ΔV_*Th*_, which captures hysteresis in the threshold region, is used to calculate Δn_*S*_ using the equation Δn_*S*_ = (*C*_ox_ ΔV*_Th_*)/*e* where *C*_*ox*_ represents the capacitance of the 90 nm SiO_2_ (38.37 × 10^–9^ F cm^–2^) and *e* is the elementary charge. To eliminate doping resulting from the programming of the bare FETs, Δn*_S,Bare_* is subtracted from Δn_*S*_ and reported in Figure 5c. All Δn_*S*_ values are tabulated in Tables S5 and S6 for bare and monolayer electrolyte FETs, respectively.

Among the measured salts, LiClO_4_ induces the largest sheet density (2 × 10^12^ cm^–2^) followed by Ca(ClO_4_)_2_ (1 × 10^12^ cm^–2^) and NaClO_4_ (0.8 × 10^12^ cm^–2^). By comparison, LiCl induces an order of magnitude less charge (0.2 × 10^12^ cm^–2^). The maximum doping density expected from a perfectly ordered monolayer electrolyte can be estimated by considering the CoCrPc packing density and ion concentration. The lateral packing density for 15C5-CoCrPc was previously measured by scanning tunneling microscopy (STM) as 0.0625 molecules/nm^2^,^11^ and the molar ratio of CoCrPc to salt is 1:1 for all salts in this study. Thus, an ion density of 0.0625 molecules/nm^2^ equates to a doping density of 6.25 × 10^12^ cm^–2^, assuming one charge is induced in the channel per cation. The perchlorate-based salts induce 12 to 32% of the theoretical maximum, while LiCl is significantly less at 3%. We originally anticipated that perhaps a divalent cation, such as Ca(ClO_4_)_2_, would induce up to two charges in the channel per cation; however, the data does not support such a conclusion.

**Figure 6:**
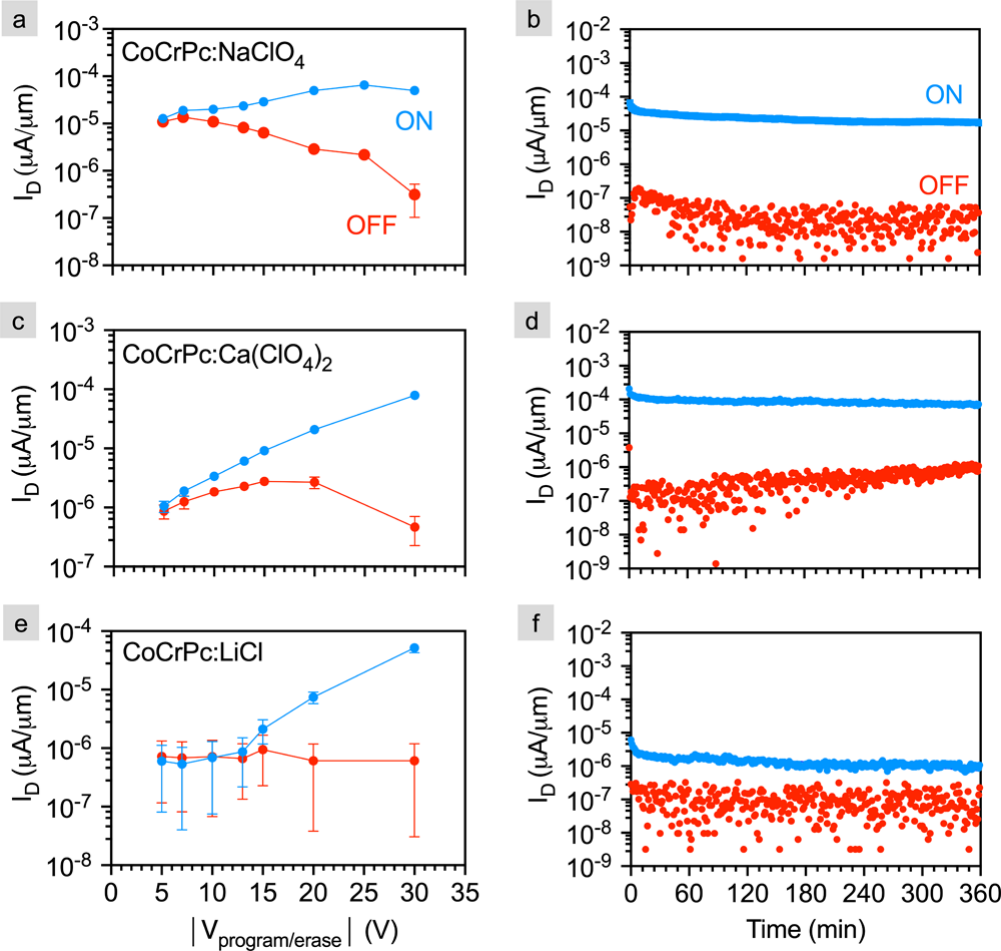
Voltage-dependent program/erase and retention measurements. ON and OFF current density, I_*D*_, as a function of programming (negative) and erase (positive) back-gate voltage, V_*program/erase*_, for (a) NaClO_4_ (2 – D1; V*_read_* = 6 V), (c) Ca(ClO_4_)_2_ (3 – D1; V*_read_* = −8 V), and (e) LiCl (4 – D1; V*_read_* = −7 V). All devices are programmed and erased for 5 min at each voltage and read for 10 s. Each data point is an average of six cycles where one cycle equals ON-read-OFF-read, and the error bars stand for one standard deviation from the mean. Retention measurements for (b) NaClO_4_ (2 – D2; V*_read_* = −2 V), (d) Ca(ClO_4_)_2_ (3 – D1; V*_read_* = −8 V), and (f) LiCl (4 – D1; V*_read_* = −5 V). All devices are programmed and erased at -/+30 V for 5 min, and the I_*D*_ is monitored every minute for 6 h.

The transfer characteristics in Figure 4 confirm channel doping with the monolayer electrolyte, and they also suggest bistability because the shift in V_*Th*_ after programming is maintained on the time scale of the measurement. Such a shift would not be maintained in a volatile electrolyte; rather, the transfer characteristics with and without programming would overlap (the data in Figure S4 shows that the initial measurements before programming do not overlap with those after programming). However, direct proof of nonvolatile switching comes from the retention measurements i.e., longer read times after either programming or erasing. Voltage-dependent program/erase and retention measurements are shown in Figure 6 for (a,b) NaClO_4_, (c,d) Ca(ClO_4_)_2_, and (e,f) LiCl. Note that LiClO_4_ is not included in this data set because we previously reported this measurement on CoCrPc:LiClO_4_.^12^

In Figure 6(a, c, e), the ON/OFF state is programmed/erased for 5 min at V_*program/erase*_ ranging from ±5 to ±30 and channel current is read for 10 s. The read voltages correspond to the maximum ON/OFF ratio on the transfer measurements in Figures 4 and S2, and therefore vary from device to device. Here, we define distinguishable ON/OFF states as separated by 2 orders of magnitude; such states are maintained for all measured salts at V_*program/erase*_ = ± 30 V. This contrasts LiClO_4_ for which only ±10 V is required to achieve two orders of ON/OFF ratio, and ±15 V to achieve over five orders of ON/OFF for the same back-gate oxide and channel thicknesses used here.^12^ Note that with 90 nm SiO_2_, and an undetermined amount of field screening by 7 to 8 layers of WSe_2_, it is not surprising that voltages of this magnitude (±10 to ±30 V) and long programming times are required to induce switching.^21^ It is straightforward to assume that top gating the device through the h-BN dielectric will reduce the required switching voltages and speed accordingly—this is work in progress.

While Figure 6a,c, and e show that ON/OFF states can be maintained for at least 10 s, Figures 6b,d, and f) monitor state retention for 6 h where I_*D*_ is read every minute. We know that the read voltages for each CoCrPc-salt system do not disturb the ON/OFF state because all read voltages are less than the program/erase voltage required to induce an appreciable difference in the ON/OFF current in Figure 6a,c, and e. For example, a read voltage of 6 V is used for CoCrPc:NaClO_4_, and the voltage required to switch the device is ±15 V (Figure 6a). While 6 h is clearly insufficient for any practical consideration of such a device, it allows us to directly compare to the state retention of LiClO_4_ published previously.^12^ NaClO_4_ and Ca(ClO_4_)_2_ demonstrate state retention for the entire 6 h measurement (i.e., more than 2 orders of magnitude ON/OFF), which is similar to the state retention of LiClO_4_. In contrast to these perchlorate-based salts, LiCl loses the 2 orders of magnitude ON/OFF current within the first few minutes of the measurement (Figure 6f). Among all the salts, LiCl also requires the largest programming/erase voltage (∼±15 V) to achieve even a discernible ON/OFF ratio (Figure 6e).

Taken together, these data suggest that monolayer electrolyte with LiClO_4_ is the best performer, with a minimum switching voltage of ±15 V to achieve an ON/OFF ratio of 10^5^, ± 10 V to achieve an ON/OFF ratio of 10^2^, and at least 6 h state retention. It is possible that the switching voltages scale with the energy barriers encountered by the different cations as they pass through the crowns. DFT calculations predict room temperature energy barriers of 0.32, 0.72, and 1.05 eV for Li^+^, Ca^2+^ and Na^+^ respectively, and when an electric field of 0.5 V/Å is applied, these barriers decrease to 0.0, 0.63, and 0.64 eV.^14^ Thus, predictions suggest that Li^+^ will have the lowest barrier to switching, while Na^+^ and Ca^2+^ will have larger, nearly equivalent barriers. It is not possible to quantitatively compare the energy barriers predicted by DFT to the barriers encountered experimentally because the voltage, which is applied to the backgate, generates an electric field that drops first through 90 nm of SiO_2_ and is then screened by several layers of heavily doped WSe_2_. After accounting for those losses, it is the remaining, unmeasured, field that reaches the monolayer electrolyte and induces the switching. However, assuming that the losses are similar from device to device, we can take the minimum voltage to program and erase the devices as a proxy for the energy required for the cation to the switch through the crown and make a qualitative assessment of the trend in energy barriers as a function of the identity of the cation. Experimentally, LiClO_4_ requires the lowest switching voltages (±10 to ±15 V) while both NaClO_4_ and Ca(ClO_4_)_2_ require higher switching voltages (±30 V), meaning that the experimental trend agrees with the theoretical prediction. Above, we hypothesized that the divalent cation, Ca^2+^, would induce more charge than the monovalent cation; however, the data in Figure 5c show that they induce similar charge densities. Although speculative, these data suggest that the doping density may be less sensitive to valency than to the barrier height to switching.

While varying energy barriers to switching could possibly explain the data for the three perchlorate based salts, it does not explain why the ON/OFF ratio and state retention for LiCl are so poor compared to LiClO_4_ since they both share the same cation. Moreover, recall that LiI, another Li-based salt, was excluded from consideration based on the poor quality of the monolayer electrolyte deposition, shown in Figure 1e. Together, these results suggest that the anion likely plays an important role in both monolayer electrolyte deposition and electrical performance, and more specifically, the best performers all share ClO_4_^–^ as the common anion. One possible explanation as to why ClO_4_^–^ based salts may perform better than those with I^–^ and Cl^–^ relates to charge delocalization. Larger, bulkier anions tend to coordinate less strongly to cations due to delocalized negative charge—a property that has been well studied by the energy storage community to promote Li^+^ transport within battery electrolytes.^22^ In the series of anions studied here, we can expect charge delocalization to increase as Cl^–^ < I^–^ < ClO_4_^–^, meaning that ClO_4_^–^ has the weakest interaction with Li^+^, favoring solvation in the crown ether. Thus, it is possible that the affinity of Li^+^ for Cl^–^ and I^–^ prohibit its function in the monolayer electrolyte because these anions disfavor solvation of Li^+^ in the crown, which is required for switching. If true, it is possible that an anion ion such as bistriflimide (TFSI^–^) with even more strongly delocalized charge than ClO_4_^–^ could further improve device performance.^23^

## Conclusions

15C5-CoCrPc combined with five different salts in a 1:1 molar ratio are studied as possible monolayer electrolytes for nonvolatile memory. Surface characterization of the monolayer electrolyte on WSe_2_ flakes is measured with AFM, and electrical characterization is performed on WSe_2_ FETs capped with h-BN. Among the three solvents measured, the best casting solvent is identified as ethanol. Among the salts studied (LiClO_4_, NaClO_4_, Ca(ClO_4_)_2_, LiI and LiCl), LiI was excluded from further investigation because a homogeneous deposition could not be achieved on WSe_2_. Specifically, the roughness, as measured by AFM, for 15C5-CoCrPc:LiI exceeds that of the other monolayer electrolytes by more than a factor of 2. All remaining salts, when combined with 15C5-CoCrPc and capped with h-BN, show bistability on WSe_2_ FETs. The change in charge density is estimated by threshold voltage shifts, and all perchlorate-based salts give rise to a charge density on the order of 10^–12^ cm^–2^. It is possible that  based salts perform better than Cl^–^ and I^–^ because of the delocalized charge favors dissociation from the cation and coordination in the cavity of the crown. Among the perchlorate-based salts, the minimum voltage required to switch the device is the monolayer electrolyte that incorporates LiClO_4_, followed by NaClO_4_ and Ca(ClO_4_)_2_ that require similar switching voltages. This observation is qualitatively consistent with predictions from DFT of energy barriers to Li^+^, Na^+^ and Ca^2+^ passing through the cavity of 15-crown-5 under an applied electric field.^14^ State retention with at least 2 orders of magnitude ON/OFF is measured for 6 h (maximum time measured) for monolayer electrolytes with LiClO_4_, NaClO_4_ and Ca(ClO_4_)_2_, while LiCl retention is lost within the first 10 min.

Building on the basic findings of this study, the results suggest the possibility of having multiple states in a one transistor, nonvolatile memory device by engineering energy barriers with different cations within 15C5-CoCrPc. Additionally, literature shows that larger crown ethers (e.g., 18C6 and 21C7) can bind with larger cations such as K^+^ and Cs^+^,^16^ and it is reasonable to assume the energy barriers for those cations to pass through larger cavities would also vary from one ion–molecule pair to another. This would expand the pool of material combinations for potential assembly of a multibit storage device. In such a device, multiple energy barriers to switching could lead to discrete resistance states akin to multibit charge trap flash memory, but with the target of a lower operating voltage.

## Methods

### Device Fabrication and Contact-Mode Cleaning

Few-layer WSe_2_ was mechanically exfoliated from its bulk crystal (2D Semiconductor) by the Scotch tape method, transferred onto a 90 nm SiO_2_/*p*-type Si substrate (Graphene Supermarket, resistivity 0.001–0.005 ohm-cm), and cleaned with acetone and isopropanol (IPA). WSe_2_ flakes with uniform thickness (∼3–8 nm) were selected by optical microscopy (Zeiss Axio); flake thickness was measured using AFM (Peakforce Tapping mode, Bruker Dimension Icon) using a Si_3_N_4_ ScanAsyst-air tip (0.4 N/m). The AFM was housed inside an Ar-filled glovebox with O_2_ and H_2_O < 2 ppm. Flakes were annealed at 240 °C for 30 min to remove air bubbles and wrinkles.^24−26^

Devices, with channel length ranging from 2–3 μm and width between 3–13 μm, were patterned with electron-beam lithography (Raith e-LINE) using PMMA-950-A4 resist (MicroChem, 4000 rpm for 1 min; 175 °C for 5 min) and developed for 1 min in methyl isobutyl ketone (MIBK)/IPA (1:3 by volume) followed by a 1 min IPA rinse. 3 nm/17 nm of Ti/Au (20 nm combined) was deposited by e-beam evaporation (Plassys Electron Beam Evaporator MEB 550 S) at a base pressure <1 × 10^–6^ Torr. Devices were soaked in acetone overnight at room temperature. The S/D contacts were kept thin (∼20 nm) near the channel to facilitate successful h-BN transfer by minimizing the height difference between the channel and contacts. However, thicker contacts were required far away from the channel to provide robust landing pads for the measurement probes to ensure good electrical contact. Thus, a second round of lithography was performed to deposit thicker contacts (5 nm/145 nm Ti/Au) connected to the thinner contacts near the channel.

Despite a standard cleaning procedure, 1–2 nm of polymer resist remains on the channels of 2D devices fabricated using EBL, and we previously showed this residue can be effectively removed using contact-mode AFM with SCM-PIT-V2 tips (Bruker Nano, 3 N/m).^19^ Removing this residue is especially important when depositing a monolayer thick film because the residue itself approximates the thickness of the monolayer electrolyte. Topology measurements were made before and after the AFM cleaning step to confirm residue removal.

### Electrolyte Deposition and h-BN Flake Transfer

Monolayer electrolyte deposition, annealing and h-BN flake transfer occurred in an Ar-filled glovebox. The electrolyte was prepared following the same procedure as described in our previous publications.^11−13^ In short, 46 μL of CoCrPc (13 mg/L) was drop-cast onto the substrate (∼1 × 1 cm) with a micropipette, followed by annealing at 240 °C for 30 min. AFM scans were performed to characterize the surface of the CoCrPc on the WSe_2_ channels. LiCl (anhydrous, ≥ 99.9% trace metal basis), NaClO_4_ (anhydrous, ≥ 99.9% trace metal basis), Ca(ClO_4_)_2_ · 4H_2_O (99.9%), and ethanol (99.5% anhydrous ethanol) were purchased from Sigma-Aldrich and used as received without any further purification. To deposit the salt solution on the same substrate (∼1 × 1 cm), 19/55/140 μL of LiCl/NaClO_4_/Ca(ClO_4_)_2_ (1 mg/L for all salt solutions) in ethanol was drop-cast onto the CoCrPc coated FETs. These mass concentrations correspond to molar ratios of 1:1 for CoCrPc to salt, and a crown ether to salt ratio of 4:1. Samples were annealed at 180 °C for 30 min.

For the FETs, the monolayer electrolyte was capped with few-layer (∼10–15 nm) h-BN (HQ Graphene). h-BN was exfoliated using the same method as WSe_2_, and flakes of 10–15 nm were picked up by a polycarbonate/polydimethylsiloxane (PC/PDMS) stamp, aligned over the channel using an optical microscope (Leica Camera AG), and pressed onto the substrate using a micromanipulator. The stack was then heated to 185 °C to release the PC from the PDMS, leaving the PC on the substrate. The residual PC was removed by immersing the substrate in chloroform at room temperature (∼25 °C) for 20 min. Delamination of the h-BN was not observed. Finally, the h-BN-capped devices were transferred via Ar-filled load-lock to the vacuum probe station for electrical measurements with no exposure to air.

### Electrical Characterization

All electrical measurements were conducted in a Lakeshore vacuum probe station (CRX-VF) using a Keysight B1500A semiconductor parameter analyzer. All devices were measured at room temperature (296 K) and a high vacuum level at 10^–6^ Torr. Drain-source voltage (V_*DS*_) equaled 500 mV for all devices except LiClO_4_, which is 20 mV for Device 1 and 100 mV for Device 2.

## Supporting Information Available

Bistable electrical switching using a Crown ether-based monolayer electrolyte on WSe_2_ field-effect transistors with various salts (PDF)
