# Supplementary material for: Bistable Electrical Switching Using a Crown Ether-Based Monolayer Electrolyte on WSe2 Field-Effect Transistors with Various Salts
Source: ACS Appl Eng Mater. 2025 Jan 30;3(2):494–501. doi: 10.1021/acsaenm.4c00799 (PMC11877525; doi:10.1021/acsaenm.4c00799)
Supplement: Supplementary file 1 — em4c00799_si_001.pdf [file em4c00799_si_001.pdf]

Supporting Information
Bistable Electrical Switching using a Crown Ether-based Monolayer Electrolyte on WSe_2 Field-effect Transistors with Various Salts

Huiran Wang,[†] Shubham Sukumar Awate,[†] and Susan K. Fullerton-Shirey[*,†,‡,¶]

†*Department of Chemical and Petroleum Engineering, University of Pittsburgh, Pittsburgh, Pennsylvania 15260, United States*

‡*Department of Electrical and Computer Engineering, University of Pittsburgh, Pittsburgh, Pennsylvania 15260, United States*

¶*Current address: 3700 O'Hara Street, Pittsburgh, Pennsylvania 15213, United States*

E-mail: fullerton@pitt.edu

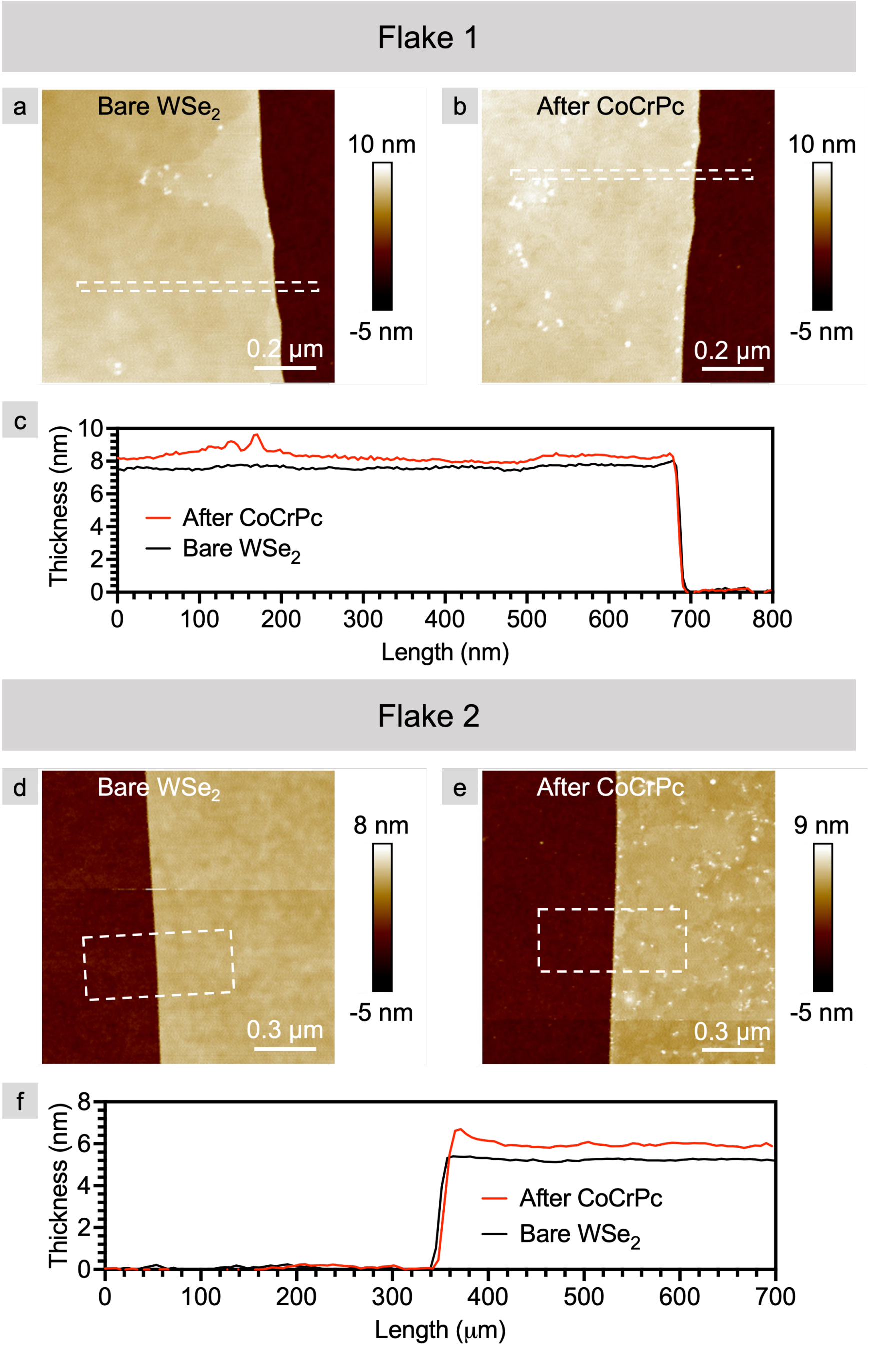

Figure S1: AFM scan **(a, d)** before and **(b, e)** after CoCrPc deposition on two different WSe_2 flakes. The white boxes in **(a, d)** and **(b, e)** correspond to 13 and 50 linescans, respectively averaged in **(c, f)**.

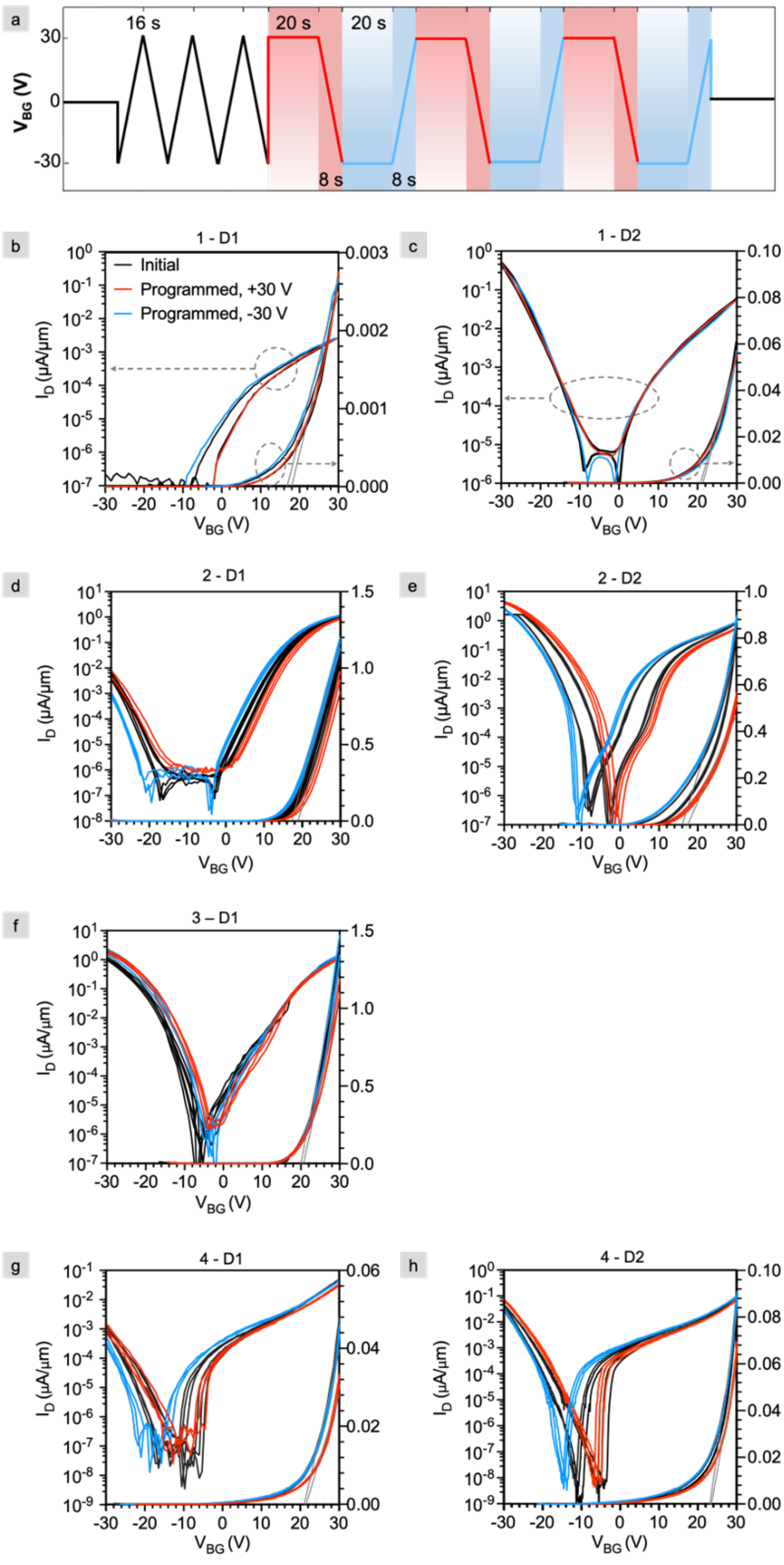
a
16 s
20 s
20 s
8 s
8 s
30
0
-30
V_BG (V)
b
1 - D1
Initial
Programmed, +30 V
Programmed, -30 V
I_D (µA/µm)
V_BG (V)
c
1 - D2
d
2 - D1
e
2 - D2
f
3 – D1
g
4 - D1
h
4 - D2

Figure S2: Bare WSe_2 FET transfer measurements with (programmed) and without (initial) programming. Measurements are repeated three times except $1-\text{D1}$ and $1-\text{D2}$ (only one measurement). **(a)** Backgate voltage (V_{BG}) sequence and resulting drain current (I_D) measurements for **(b)** $1-\text{D1}$ and **(c)** $1-\text{D2}$, **(d)** $2-\text{D1}$ and **(e)** $2-\text{D2}$, **(f)** $3-\text{D1}$, and **(g)** $4-\text{D1}$ and **(h)** $4-\text{D2}$. To improve clarity, only the n-branch is shown on the linear scale. The programming time is 20 s for all devices except $1-\text{D1}$ which is programmed for 5 mins, and $1-\text{D2}$ which is programmed for 0.1 s. Sweep rate is ~ 7.5 V/s for all devices except $1-\text{D2}$ which is sweep at ~ 5 V/s. The gray lines are an extrapolation of the linear fits to zero current ($I_D = 0\ \mu A/\mu m$). The data for $1-\text{D2}$ is from Liang, et al.[1]

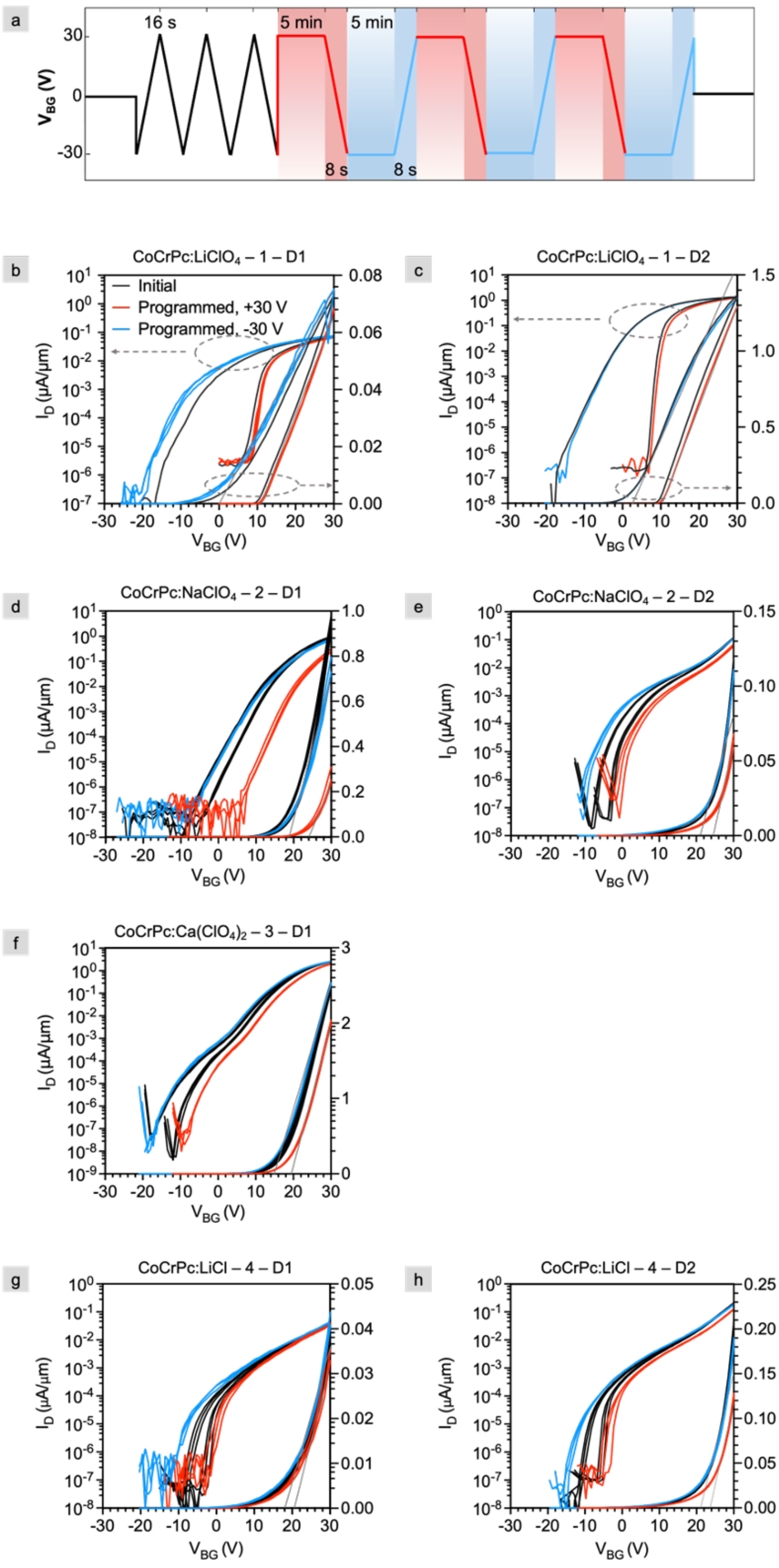
a
16 s
5 min
5 min
8 s
8 s
V_BG (V)
b
CoCrPc:LiClO4 – 1 – D1
Initial
Programmed, +30 V
Programmed, -30 V
I_D (μA/μm)
V_BG (V)
c
CoCrPc:LiClO4 – 1 – D2
d
CoCrPc:NaClO4 – 2 – D1
e
CoCrPc:NaClO4 – 2 – D2
f
CoCrPc:Ca(ClO4)2 – 3 – D1
g
CoCrPc:LiCl – 4 – D1
h
CoCrPc:LiCl – 4 – D2

Figure S3: Monolayer electrolyte WSe_2 FET transfer measurements with (programmed) and without (initial) programming. Measurements are repeated three times except the initial measurements (i.e., without programming) of 1 − D1 and 1 − D2. **(a)** V_{BG} sequence and resulting I_D measurements for CoCrPc:$LiClO_4$: **(b)** 1 − D1 and **(c)** 1 − D2, CoCrPc:$NaClO_4$ **(d)** 2 − D1 and **(e)** 2 − D2, CoCrPc:$Ca(ClO_4)_2$ **(f)** 3 − D1, and CoCrPc:LiCl **(g)** 4 − D1 and **(h)** 4 − D2 (only 2nd, 3rd, and 4th data entry in Table S2 & S4 plotted here). For clarity, only n-branch is shown on the linear scale, similar to the bare devices. The programming time is 5 mins for all devices except 1 − D2 which is programmed for 1 s. Sweep rate is ∼ 7.5 V/s for all devices except 1 − D2 which is sweep at ∼ 10 V/s. The gray lines are an extrapolation of the linear fits to zero current ($I_D = 0$ μA/μm). The data for 1 − D1 is from Liang, et al.[1]

Table S1: Sub-threshold voltages (V_S) for the n-branch of bare, WSe_2 FETs in the initial and programmed states.

Bare WSe_2 Device	State	V_S ($- \rightarrow +30$ V)	V_S ($+ \rightarrow -30$ V)	ΔV_S	$\Delta V_{S,Avg}$
1 − D1*	Initial	−3.8	0.3	4.1	N/A
	Programmed	−5.4	0.7	6.1	N/A
1 − D2	Initial	1.4	−0.3	1.7	N/A
	Programmed	0.6	−0.2	−0.8	N/A
2 − D1	Initial	2.2	3.3	1.1	1.0 ± 0.4
		1.8	2.3	0.5	
		1.4	2.7	1.3	
	Programmed	0.4	4.6	4.2	4.2 ± 0.1
		0.1	4.3	4.2	
		−0.4	3.7	4.1	
2 − D2	Initial	−3.5	1.3	4.8	5.1 ± 0.4
		−3.9	1.0	4.9	
		−4.3	1.2	5.5	
	Programmed	−6.0	3.0	9.0	8.7 ± 0.3
		−6.6	2.0	8.6	
		−6.9	1.6	8.5	
3 − D1	Initial	−0.3	0.8	1.1	1.3 ± 0.5
		−2.1	−1.1	1.0	
		−2.7	−0.9	1.8	
	Programmed	−0.6	0.3	0.9	1.8 ± 1.4
		−1.3	2.1	3.4	
		0.4	1.4	1.0	
4 − D1	Initial	−9.1	−4.3	4.8	4.4 ± 0.4
		−9.9	−5.7	4.2	
		−10.4	−6.2	4.2	
	Programmed	−13.6	−5.1	8.5	7.7 ± 0.7
		−13.9	−6.8	7.1	
		−14.5	−6.9	7.6	
4 − D2	Initial	−8.3	−2.9	5.4	5.1 ± 0.2
		−9.5	−4.4	5.1	
		−10.1	−5.2	4.9	
	Programmed	−12.3	−4.2	8.1	7.8 ± 0.3
		−13.0	−5.2	7.8	
		−13.6	−6.1	7.5	

Data are fit to a line over more than two decades in current and all R^2 values range from 0.87 to 1.00. The current density for reading V_S is 1×10^{-5} μA/μm for all devices except Devices 1 − D1, 4 − D1, and 4 − D2 for which the current density is 1×10^{-6} μA/μm. Error represents one standard deviation from the mean.

* The data for 1 − D1 is from Liang, et al.[1]

Table S2: Sub-threshold voltage (V_S) for the n-branch of WSe_2 FETs with four monolayer electrolytes in the initial and programmed states.

Salt	State	V_S ($- \to +30$ V)	V_S ($+ \to -30$ V)	ΔV_S	$\Delta V_{S,Avg}$
$LiClO_4$ (1 − D1)*	Initial	−11.9	7.0	18.9	N/A
	Programmed	−14.9	8.6	23.5	24.1 ± 0.6
		−15.7	9.0	24.8	
		−15.9	8.3	24.2	
$LiClO_4$ (1 − D2)	Initial	−15.4	6.5	21.9	N/A
	Programmed	−14.3	7.2	21.5	N/A
$NaClO_4$ (2 − D1)	Initial	−3.4	−0.1	3.3	3.5 ± 0.3
		−3.9	−0.5	3.4	
		−4.3	−0.4	3.9	
	Programmed	−3.7	8.5	12.2	13.1 ± 0.9
		−4.1	9.1	13.2	
		−4.6	9.3	13.9	
$NaClO_4$ (2 − D2)	Initial	−5.4	−1.8	3.6	3.6 ± 0.0
		−5.9	−2.3	3.6	
		−6.1	−2.5	3.6	
	Programmed	−8.3	0.0	8.3	8.9 ± 0.2
		−9.6	−0.7	8.9	
		−10.4	−1.7	8.7	
$Ca(ClO_4)_2$ (3 − D1)	Initial	−13.5	−7.9	5.6	5.1 ± 0.5
		−13.7	−8.7	5.0	
		−13.8	−9.2	4.6	
	Programmed	−14.0	−5.6	8.4	8.5 ± 0.2
		−14.1	−5.3	8.8	
		−14.0	−5.7	8.3	
LiCl (4 − D1)	Initial	−11.1	−8.4	2.7	3.6 ± 0.9
		−5.7	−1.3	4.4	
		−6.7	−2.3	4.4	
		−7.7	−2.8	4.9	
		−6.0	−2.7	3.3	
		−6.5	−3.5	3.0	
		−7.0	−4.1	2.9	
		−5.4	−1.1	4.3	
		−6.4	−3.9	2.5	
	Programmed	−13.0	−4.5	8.5	8.3 ± 1.2
		−9.8	−0.3	9.5	
		−10.2	−0.9	9.3	
		−10.8	−2.7	8.1	
		−9.2	−1.3	7.9	
		−9.4	−2.1	7.3	
		−10.0	−2.4	7.6	
		−9.6	0.3	9.9	
		−9.0	−2.7	6.3	
LiCl (4 − D2)	Initial	−9.0	−4.7	4.3	4.2 ± 0.1
		−9.6	−5.5	4.1	
		−10.2	−6.1	4.1	
	Programmed	−12.9	−3.0	9.9	9.3 ± 0.5
		−13.7	−4.6	9.1	
		−14.6	−5.7	8.9	

Data are fit to a line over two decades in current and R^2 values for all the fittings range from 0.89 to 1.00. The current density for reading V_S is 1×10^{-6} μA/μm for all devices except 1 − D1 for which the current density is 1×10^{-5} μA/μm. Error represents one standard deviation from the mean.

* Data of $LiClO_4$(1 − D1) is from Liang, et al.[1]

Table S3: Threshold voltages (V_{Th}) for the n-branch of bare, WSe_2 FETs in the initial and programmed states.

Bare WSe_2 Device	State	V_{Th} ($- \rightarrow +30$ V)	V_{Th} ($+ \rightarrow -30$ V)	ΔV_{Th}	$\Delta V_{Th,Avg}$
1 − D1*	Initial	16.7	17.9	1.3	N/A
	Programmed	16.5	18.1	1.6	N/A
1 − D2	Initial	21.7	21.3	−0.4	N/A
	Programmed	21.7	21.1	−0.6	N/A
2 − D1	Initial	18.1<br>18.0<br>17.8	18.7<br>18.4<br>18.3	0.6<br>0.4<br>0.5	0.54 ± 0.1
	Programmed	17.7<br>17.3<br>17.0	18.9<br>18.6<br>18.2	1.2<br>1.3<br>1.2	1.2 ± 0.0
2 − D2	Initial	17.3<br>17.6<br>17.5	18.8<br>18.8<br>18.7	1.5<br>1.2<br>1.2	1.3 ± 0.2
	Programmed	16.1<br>16.1<br>16.0	17.8<br>17.5<br>17.3	1.7<br>1.4<br>1.3	1.4 ± 0.2
3 − D1	Initial	19.9<br>20.0<br>20.2	20.2<br>20.0<br>20.4	0.3<br>0.0<br>0.2	0.18 ± 0.1
	Programmed	20.1<br>20.1<br>19.9	20.2<br>20.6<br>20.2	0.1<br>0.5<br>0.3	0.30 ± 0.2
4 − D1	Initial	21.0<br>21.1<br>21.2	21.3<br>21.2<br>21.4	0.3<br>0.1<br>0.2	0.19 ± 0.1
	Programmed	20.1<br>20.2<br>20.3	21.5<br>21.6<br>21.5	1.4<br>1.4<br>1.2	1.3 ± 0.1
4 − D2	Initial	23.2<br>22.9<br>23.0	23.5<br>23.5<br>23.5	0.3<br>0.6<br>0.5	0.43 ± 0.1
	Programmed	22.6<br>22.5<br>22.6	23.5<br>23.5<br>23.3	0.9<br>1.0<br>0.7	0.84 ± 0.2

I_D was fit on a linear scale at least 4 V in V_{BG} with $R^2 = 0.95$. Error represents one standard deviation from the mean.
* The data for 1 − D1 is from Liang, et. al.[1]

Table S4: Threshold voltages (V_{Th}) for the n-branch of WSe_2 FETs with four monolayer electrolytes in the initial and programmed states

Salt	State	V_{Th} ($- \rightarrow +30$ V)	V_{Th} ($+ \rightarrow -30$ V)	ΔV_{Th}	$\Delta V_{Th,Avg}$
$LiClO_4$ (1 − D1)*	Initial	3.2	11.4	8.3	N/A
	Programmed	1.4	11.4	10.0	
		−0.6	11.7	12.2	11.2 ± 0.7
		−0.1	11.4	11.5	
$LiClO_4$ (1 − D2)	Initial	2.2	9.1	6.9	N/A
	Programmed	2.3	10.4	8.1	N/A
$NaClO_4$ (2 − D1)	Initial	18.9	20.3	1.4	
		18.9	19.9	1.0	1.2 ± 0.2
		18.7	19.8	1.1	
	Programmed	18.5	24.0	5.5	
		18.4	24.2	5.8	5.7 ± 0.2
		18.4	24.2	5.8	
$NaClO_4$ (2 − D2)	Initial	22.4	24.1	1.7	
		22.5	24.1	1.6	1.6 ± 0.0
		22.5	24.1	1.6	
	Programmed	21.0	24.4	3.4	
		21.1	24.5	3.4	3.4 ± 0.1
		21.0	24.5	3.5	
$Ca(ClO_4)_2$ (3 − D1)	Initial	15.4	16.0	0.6	
		15.2	15.8	0.7	0.7 ± 0.0
		15.1	15.7	0.7	
	Programmed	14.8	19.3	4.4	
		14.9	19.4	4.5	4.5 ± 0.1
		14.9	19.5	4.6	
LiCl (4 − D1)	Initial	19.4	21.2	1.8	
		18.0	19.8	1.8	
		17.9	19.5	1.6	
		18.0	19.9	1.9	
		18.5	19.1	0.5	1.1 ± 0.7
		18.7	19.1	0.4	
		18.2	19.0	0.8	
		20.2	20.9	0.7	
		20.2	20.5	0.3	
	Programmed	18.4	22.4	4.0	
		18.2	20.4	2.2	
		18.1	20.3	2.2	
		18.3	20.7	2.4	
		18.1	19.9	1.8	2.1 ± 0.8
		18.3	19.9	1.6	
		18.2	20.2	2.0	
		19.9	21.3	1.4	
		19.7	20.9	1.2	
LiCl (4 − D2)	Initial	22.1	22.8	0.7	
		22.2	22.9	0.7	0.6 ± 0.0
		22.1	22.7	0.6	
	Programmed	20.6	23.5	2.9	
		20.8	23.6	2.8	2.9 ± 0.0
		20.7	23.6	2.9	

I_D was fit on a linear scale at least 4 V in V_{BG} with $R^2 = 0.95$. Error represents one standard deviation from the mean.

* Data of $LiClO_4$ (1 − D1) is taken from Liang, et. al.[1]

Table S5: Change in sheet carrier density (Δn_S, *Bare*) for the n-branch of bare, WSe_2 FETs in the initial and programmed states. $C_{OX} = 38.37 \times 10^{-9}$ F cm^{-2} (90 nm SiO_2). Mobility of the bare FETs ranges from 0.5 to 25 cm^2/Vs.

Bare WSe_2 Device	State	$\Delta n_{S,Bare}$ [cm^{-2}]
1 − D1	Initial	3.1×10^{11}
	Programmed	3.7×10^{11}
1 − D2	Initial	8.9×10^{10}
	Programmed	1.4×10^{11}
2 − D1	Initial	$1.3 \times 10^{11} \pm 2.9 \times 10^{10}$
	Programmed	$3.0 \times 10^{11} \pm 1.1 \times 10^{10}$
2 − D2	Initial	$3.1 \times 10^{11} \pm 5.0 \times 10^{10}$
	Programmed	$3.5 \times 10^{11} \pm 4.1 \times 10^{10}$
3 − D1	Initial	$4.3 \times 10^{10} \pm 3.1 \times 10^{10}$
	Programmed	$7.3 \times 10^{10} \pm 5.9 \times 10^{10}$
4 − D1	Initial	$4.6 \times 10^{10} \pm 1.4 \times 10^{10}$
	Programmed	$3.2 \times 10^{11} \pm 2.4 \times 10^{10}$
4 − D2	Initial	$1.0 \times 10^{11} \pm 3.2 \times 10^{10}$
	Programmed	$2.0 \times 10^{11} \pm 4.1 \times 10^{10}$

Table S6: Change in sheet carrier density (Δn_S) for the n-branch of monolayer electrolyte WSe_2 FETs in the initial and programmed states. To isolate Δn_S induced by the monolayer electrolytes, $\Delta n_{S,Bare}$ from Table S5 is subtracted from Δn_S for each device. C_{OX} = 38.37 $\times 10^{-9}$ F cm^{-2} (90 nm SiO_2). Mobility of the monolayer electrolyte FETs ranges from 0.5 to 50 cm^2/Vs.

Salt	State	Δn_S [cm^{-2}]	Δn_S - $\Delta n_{S,Bare}$ [cm^{-2}]
$LiClO_4$ (1 − D1)	Initial	2.0×10^{12}	1.7×10^{12}
	Programmed	$2.7 \times 10^{12} \pm 2.7 \times 10^{11}$	$2.3 \times 10^{12} \pm 2.7 \times 10^{11}$
$LiClO_4$ (1 − D2)	Initial	1.7×10^{12}	1.6×10^{12}
	Programmed	1.9×10^{12}	1.8×10^{12}
$NaClO_4$ (2 − D1)	Initial	$2.8 \times 10^{11} \pm 5.0 \times 10^{10}$	$1.5 \times 10^{11} \pm 5.8 \times 10^{10}$
	Programmed	$1.4 \times 10^{12} \pm 4.3 \times 10^{10}$	$1.1 \times 10^{12} \pm 4.4 \times 10^{10}$
$NaClO_4$ (2 − D2)	Initial	$3.9 \times 10^{11} \pm 5.0 \times 10^{9}$	$7.8 \times 10^{10} \pm 5.1 \times 10^{10}$
	Programmed	$8.2 \times 10^{11} \pm 1.6 \times 10^{10}$	$4.8 \times 10^{11} \pm 4.4 \times 10^{10}$
$Ca(ClO_4)_2$ (3 − D1)	Initial	$1.6 \times 10^{11} \pm 2.8 \times 10^{9}$	$1.1 \times 10^{11} \pm 3.2 \times 10^{10}$
	Programmed	$1.1 \times 10^{12} \pm 1.6 \times 10^{10}$	$1.0 \times 10^{12} \pm 6.1 \times 10^{10}$
LiCl (4 − D1)	Initial	$2.6 \times 10^{11} \pm 1.6 \times 10^{11}$	$2.1 \times 10^{11} \pm 1.6 \times 10^{11}$
	Programmed	$5.0 \times 10^{11} \pm 2.0 \times 10^{11}$	$1.8 \times 10^{11} \pm 2.0 \times 10^{11}$
LiCl (4 − D2)	Initial	$1.5 \times 10^{11} \pm 9.1 \times 10^{9}$	$5.1 \times 10^{10} \pm 3.3 \times 10^{10}$
	Programmed	$6.9 \times 10^{11} \pm 8.7 \times 10^{9}$	$1.1 \times 10^{11} \pm 3.2 \times 10^{10}$

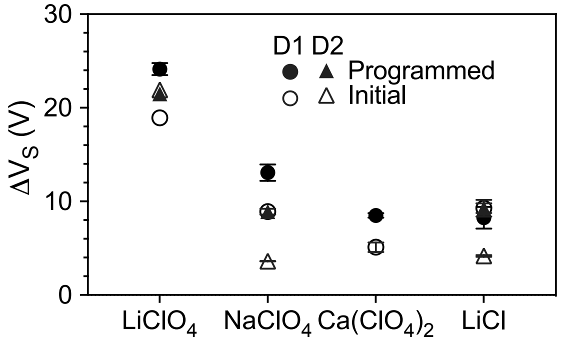

Figure S4: Summary of voltage shifts for monolayer electrolyte-coated WSe_2 FETs in the initial and programmed states. Voltage differences between the forward (−30 to +30 V) and reverse (−30 to +30 V) transfer measurements before (initial, open) and after (programmed, filled) ±30 V programming in the subthreshold voltage shift (ΔV_S) for FETs. D1 (circles) and D2 (triangles) stand for Device 1 and 2 for each salt. Most data are averaged over three ON/OFF programming cycles, as detailed above in Table S1 & S2. Error bars indicate one standard deviation from the mean.

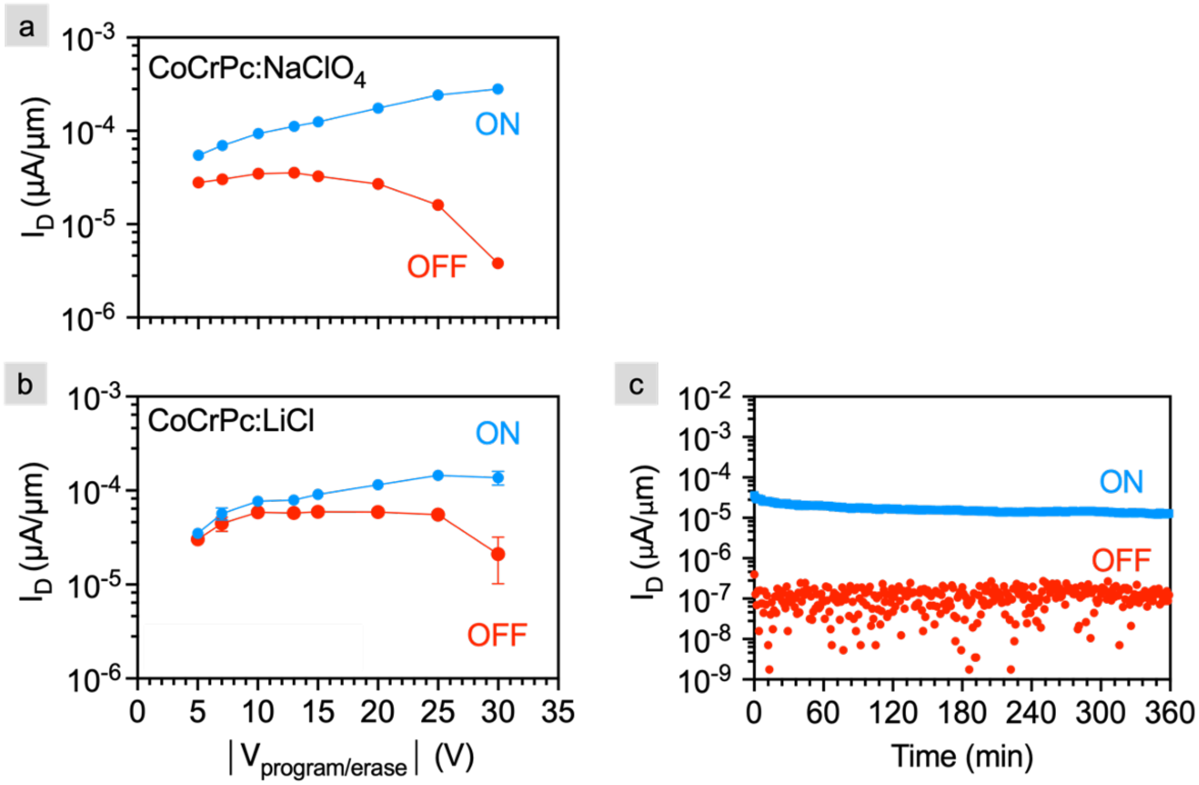

Figure S5: Voltage-dependent program/erase and retention measurements for WSe_2 FETs with ((**a**) CoCrPc:$NaClO_4$ (2 – D2; V_{read} = -2 V) and (**b**) (V_{read} = -6 V) and (**c**) CoCrPc:LiCl (4 – D2; V_{read} = -12 V), respectively. For program/erase, I_D is measured after programming and erasing, both for 5 mins, at voltages (V_{BG}) ranging from ±5 to ±30 V. Retention measurements are made by setting the ON and OFF states by programming each state for 5 minutes, and then monitoring I_D every minute for 6 hours.

References

(1) Liang, J.; Xu, K.; Wu, M.; Hunt, B. M.; Wang, W. H.; Cho, K.; Fullerton-Shirey, S. K. Molecularly Thin Electrolyte for All Solid-State Nonvolatile Two-Dimensional Crystal Memory. *Nano Letters* **2019**, *19*, 8911–8919.
